# From Gendered Diagnosis to Community Care: How Borderline Personality Disorder Organizes Community Mental Health Nursing Practice

**DOI:** 10.1111/nin.70174

**Published:** 2026-10-25

**Authors:** Rachel Galea, Alison Rodgers, Catherine MacDonald, Ayanna Robertson, Alessandra Rooney

**Affiliations:** ^1^ School of Nursing Trent University Peterborough Ontario Canada

**Keywords:** borderline personality disorder, community, feminism, mental health, nursing

## Abstract

Borderline personality disorder (BPD) is a contested diagnosis that has been criticized for pathologizing women's responses to trauma, adversity, and relational harm. This paper examines how BPD, as a gendered, historically situated, and institutionally mediated diagnostic category, shapes community mental health nursing (CMHN) practice. Using a critical conceptual analysis, it synthesizes historical, feminist, psychiatric, nursing, and Mad studies literature, combining genealogical, critical feminist, and social‐structural perspectives. The paper argues that BPD functions not only as a clinical description of symptoms but also as an institutional category that influences documentation, referral pathways, eligibility criteria, and service design. Through these processes, the diagnosis shapes clinical visibility, therapeutic relationships, service access, and continuity of care. Moving beyond explanations of stigma that focus solely on individual clinician attitudes, the analysis demonstrates how diagnostic meanings are reproduced through organizational and health system structures, contributing to forms of structural stigma that constrain relational and trauma‐informed care. The paper concludes with implications and recommendations for nursing practice, education, service delivery, and policy, including trauma‐informed and relational approaches to assessment, non‐stigmatizing documentation practices, transparent referral and eligibility processes, investment in community‐based services, and strengthened clinical supervision and workforce supports.

## 1 Introduction

### 1.1 The Problem

Borderline personality disorder (BPD) is a contested diagnosis criticized for pathologizing women's responses to violence, trauma, and relational harm by reframing contextual or adaptive responses to suffering as personality pathology (Ma and Else‐Quest 2025; Shaw and Proctor 2005). This critique is particularly relevant to nursing, where diagnostic labels shape how distress is interpreted, documented, and responded to in everyday practice.

Although BPD is diagnosed disproportionately among women in clinical settings, often at an approximate ratio of 3:1, community prevalence studies suggest a much less pronounced gender difference (Gunderson et al. 2018). This discrepancy raises important questions about how diagnostic meaning and detection vary across settings. The diagnosis is also strongly associated with histories of childhood trauma (Cattane et al. 2017) and remains marked by significant stigma within health care settings (Galea et al. 2026; Klein et al. 2026). People diagnosed with BPD are often described by nurses and other clinicians as “difficult,” “manipulative,” or “untreatable,” perceptions that can shape therapeutic relationships, clinical decision‐making, and the quality of care received (Loader 2017; McLaughlin et al. 2025).

These concerns are evident within the Canadian health care system, and similar patterns have been documented internationally, particularly in the United Kingdom, the United States, and Australia (Galea et al. 2026). In the United Kingdom, longstanding concerns about the systemic exclusion of personality disorders from mainstream mental health services led to the development of “Personality Disorder: No Longer a Diagnosis of Exclusion” (National Institute for Mental Health in England 2003). This policy sought to improve access to and support the development of mental health services for people diagnosed with personality disorders, including BPD. Despite such early policy response, inequitable access to health care, therapeutic pessimism, and stigmatizing service patterns continue to be reported internationally (Galea et al. 2026).

These issues are salient for nursing because nurses are often involved in assessment, documentation, care coordination, follow‐up, and ongoing therapeutic engagement across mental health settings (College of Nurses of Ontario 2023). BPD is highly represented in psychiatric services, with prevalence estimates of approximately 12% in outpatient settings and 22% in inpatient settings (Ellison et al. 2018). Nurses are therefore likely to encounter people diagnosed with BPD across emergency, inpatient, outpatient, and community contexts. Following brief inpatient admissions, individuals diagnosed with BPD are often quickly discharged to outpatient or community‐based services for continuing care (Biskin and Paris 2012). Consequently, community mental health nurses (CMHNs) frequently support individuals diagnosed with BPD across transitions in care and over extended periods of service involvement. As a result, CMHNs are often positioned at the intersection of diagnostic practices, risk management processes, treatment decisions, and ongoing therapeutic relationships, making them uniquely situated to observe how the diagnosis shapes experiences of care and access to services.

This paper traces how the BPD diagnosis organizes CMHN work, shaping clinical visibility, relational engagement, eligibility, and care continuity, rather than functioning solely as an individual clinical descriptor. It extends feminist and mad‐studies critiques into nursing by treating diagnosis as an institutional organizing mechanism that reproduces gendered and managerial logics. Finally, it offers practical recommendations aimed at shifting responsibility from individual clinicians toward institutional systems.

### 1.2 Diagnosis as Institutional and Clinical Processes

A central premise of this paper is that diagnoses do not enter nursing practice as neutral or purely descriptive accounts of distress. Rather, diagnoses are taken up through institutional and clinical processes that organize how patients are seen, assessed, documented, and treated. This paper examines how BPD becomes active in CMHN as diagnostic meanings are carried through texts, translated into risk and eligibility decisions, and used to shape referrals and care planning.

We are particularly interested in how nurses' everyday practices are shaped by institutional processes that may obscure histories of trauma, gendered harm, and structural violence. In this sense, BPD enters mental health nursing in community contexts not only as diagnostic knowledge, but also as an organizing category activated under conditions that prioritize accountability, manageability, and service flow. Rankin and Campbell's (2006) analysis of Canadian health care reform is useful here, as it illuminates how nursing work is coordinated through managerial, text‐based, and efficiency‐oriented systems that shape what nurses notice, document, and do in clinical settings. In practice, these institutional processes may prioritize risk, compliance, and symptom management, often displacing relational, contextual, and gendered understandings of distress. The diagnosis can activate sequences of action, including heightened surveillance, boundary‐setting strategies, restricted service access, or referral exclusion (Klein et al. 2022; Sulzer 2015). These responses may be treated as clinically necessary, yet they are also institutionally organized.

Although a substantial body of literature has examined stigmatizing attitudes toward individuals diagnosed with BPD among nurses and other health care providers (Dickens et al. 2016; Loader 2017; McKenzie et al. 2022; McLaughlin et al. 2025; Stiles et al. 2023), less attention has been paid to how the diagnosis itself operates through institutional and organizational processes to shape CMHN practice. A focus on provider attitudes alone risks obscuring how stigma is reproduced through the arrangements that structure the conditions under which care is delivered and experienced, rather than arising solely from individual prejudice (Desai et al. 2021; Dickens et al. 2016; Galea et al. 2026; Klein et al. 2022).

### 1.3 Community Mental Health Nursing

In this paper, CMHN refers broadly to nursing work occurring outside inpatient units, including outpatient clinics, outreach programs, community services, and case management contexts (Canadian Federation of Mental Health Nurses 2023). At its best, CMHN practice is relational, longitudinal, and embedded in systems of referral, follow‐up, documentation, and care coordination across fragmented health systems (Canadian Federation of Mental Health Nurses 2023; Hegedüs et al. 2025). Their work commonly includes direct care, education, advocacy, case management, documentation, and collaboration with interprofessional teams. As a result, CMHNs are closely involved in how individuals diagnosed with BPD navigate and experience mental health services, and treatment options made available to them.

In this context, a diagnosis of BPD can shape how distress is interpreted, how risk and urgency are assessed, and how therapeutic relationships are negotiated across repeated encounters (Loader 2017; Navarre 2025). By treating BPD as an organizing diagnostic category, this paper shows how everyday CMHN practice is shaped by broader historical, gendered, and institutional conditions.

### 1.4 Aims

This discussion paper asks how BPD's historical formation continues to influence contemporary CMHN practice and how the diagnosis functions as a category that organizes clinical visibility, relational care, service access, and continuity of care. It has two aims: (1) to trace the genealogy of BPD and explain why the diagnosis matters for CMHN, and (2) to analyze how BPD functions as an institutionally organized diagnostic category that shapes nursing practice through clinical, organizational, and professional processes.

This paper does not seek to adjudicate the validity of BPD as a diagnosis. Rather, using a critical conceptual lens, it treats BPD as both a clinical diagnosis and an institutional category that coordinates decision‐making across care settings and shapes pathways to treatment and support. It concludes with recommendations for practice, education, policy, and service delivery that promote relational accountability, reduce diagnostic stigma, and strengthen quality of care.

### 1.5 Conceptual and Methodological Approach

Rather than presenting new empirical data, this critical conceptual analysis synthesizes historical, psychiatric, feminist, nursing, and Mad studies literature to examine how BPD has been constructed and how its meanings shape contemporary CMHN practice. Literature searches were purposive, drawing on peer‐reviewed and gray literature related to BPD, nursing, health care, and community mental health settings. As a conceptual analysis, the review was designed to foreground these domains and does not attempt to comprehensively capture all literature relevant to BPD, mental health, or psychiatric diagnosis.

The analysis integrates four complementary perspectives. A genealogical approach traces how particular meanings attached to BPD became stabilized within psychiatric discourse, emphasizing the diagnosis as historically situated rather than a fixed or self‐evident clinical category (Jones 2023). Critical feminist analysis highlights how psychiatric knowledge has historically pathologized emotional expression, anger, behavior, and relational suffering, particularly when associated with women, shaping both diagnostic practices and clinical interpretation (Chesler 1972; Ma and Else‐Quest 2025; Shaw and Proctor 2005). A social‐structural lens examines how these meanings are reproduced through institutional practices, including documentation, referral criteria, risk assessment, eligibility rules, and service pathways (Rankin and Campbell 2006). Mad Studies is an interdisciplinary field and activist knowledge project grounded in the scholarship, histories, cultural production, and political organizing of people who identify as Mad, psychiatric survivors, service users, or consumers (LeFrançois et al. 2013). It examines sanism and psychiatric power while centering lived experience, refusal, resistance, and non‐pathologizing ways of understanding distress (Burstow 2015; LeFrançois et al. 2013; Redikopp 2018). Although Mad Studies is survivor‐led, its concerns intersect with broader critical scholarship that challenges coercive, gendered, and exclusionary psychiatric practices (Chesler 1972; Shaw and Proctor 2005).

Although complementary, these perspectives are not theoretically interchangeable. Genealogical analysis historicizes diagnostic truth claims but does not necessarily privilege the experiences of those subjected to them (Jones 2023). Critical feminist analysis foregrounds gendered relations of power but risks treating women as a homogeneous category if differences across social locations are insufficiently considered (Crenshaw 1991; Collins 2000). Social‐structural analysis traces how texts and organizational processes coordinate practice (Rankin and Campbell 2006), but may underemphasize refusal, survivor knowledge, and epistemic resistance (Redikopp 2018). Mad Studies more directly challenges psychiatric and professional authority, including the scholarly authority through which conceptual analyses are produced (Burstow 2015; LeFrançois et al. 2013). Rather than resolving these differences or combining the perspectives into a unified framework, we maintain their distinctive contributions and use points of convergence and divergence as analytic resources throughout the analysis. These perspectives are adopted reflexively, acknowledging both the value and the limitations of theory‐driven analysis and our positionality as scholar‐practitioners attentive to gendered and institutional dimensions of mental health care.

## 2 Gendered Psychiatry and Nursing

### 2.1 Genealogy of the Diagnostic Classification of BPD

The diagnosis of BPD is rooted in historical, social, and gendered processes that continue to shape CMHN practice. Rather than emerging as a neutral or self‐evident diagnostic entity, BPD can be genealogically traced through psychiatry's gendered interpretations of women's distress (Jones 2023; Shaw and Proctor 2005). A critical feminist reading of this history makes visible how psychiatric discourse has participated in governing women's bodies and behaviors through diagnostic categories. This history matters not only because it illuminates the origins of the diagnosis but also because it shows how social, cultural, and economic systems have contributed to diagnostic frameworks that interpret women's emotions as signs of pathology (Valero et al. 2025).

A key precursor to BPD is hysteria, traced in Western accounts back millennia, which historically framed women's psychological disturbance allegedly caused by movements of the uterus (Tasca et al. 2012). Although explanatory models shifted over time, hysteria functioned for centuries as a way of interpreting women's distress through bodily, moral, and social deviance. In the pre‐modern era, so‐called hysterical behaviors were often attributed to women whose conduct challenged dominant social and moral norms (Tasca et al. 2012). Emotional intensity, sexual expression, anger, nonconformity, and relational disruption were therefore not merely observed as signs of suffering but coded as evidence of disorder. As Shaw and Proctor (2005) argue, such characteristics formed part of a patriarchal interpretive framework through which difficult or disruptive female behavior was rendered intelligible as pathology. What mattered was not simply distress itself, but whether that distress aligned with or violated gendered expectations.

This dynamic also took overtly punitive forms. During the Middle Ages, women whose behavior fell outside accepted patriarchal norms could be demonized as witches, with emotional suffering, social marginality, and resistance to authority interpreted through religious and misogynistic logics (Tasca et al. 2012). Although these practices differ from modern psychiatric diagnosis, they illustrate a persistent pattern in which women's suffering, particularly when it challenged dominant norms, was recast as individual, moral, or spiritual defect rather than socially situated distress.

This pattern persisted in modern psychiatry. Chesler's (1972) *Women and Madness* documents how women who deviated from expected gender roles were medicalized and institutionalized. Drawing on interviews with women institutionalized between 1950 and 1970, Chesler described how women were pressured to self‐admit by husbands, male psychiatrists, and parents after being labeled insane for speaking out against abuse, experiencing postpartum distress, or challenging gender norms. Although historically removed from hysteria, these accounts reveal an important continuity: women's self‐advocacy, anger, and resistance could still be reframed as evidence of mental instability (Dorfman and Reynolds 2023; Ma and Else‐Quest 2025).

The term “borderline” emerged in psychoanalytic discourse to label patients seen as between psychosis and neurosis, often those considered difficult to classify or treat (Leichsenring et al. 2024; Gunderson et al. 2018). During the 1970s, the rise of descriptive psychiatry and psychopharmacology shifted psychiatric classification toward more standardized diagnostic categories (Gunderson 2009), leading to BPD being introduced as a formal diagnosis in DSM‐III in 1980, and remaining a recognizable diagnostic category across subsequent DSM editions (American Psychiatric Association 1980). Its inclusion in the DSM should not be mistaken for the discovery of a previously hidden disorder. Rather, it marked the stabilization of a category already shaped by psychiatric negotiation and inherited meanings about emotional instability, dependency, and relational volatility (Horwitz 2023). These are not incidental features; they are characteristics that have long been culturally coded in gendered ways.

BPD is formally applied through psychiatric frameworks such as the Diagnostic and Statistical Manual of Mental Disorders DSM‐5‐TR (DSM‐5‐TR), which defines BPD with criteria of instability in relationships, self‐image, affect regulation, impulsivity, and suicidal behavior (American Psychiatric Association 2022). Although these criteria appear descriptive, their uptake is highly interpretive and subjective within clinical and organizational settings, settings which transform them into actionable categories that structure care.

Historical meanings continue to shape contemporary diagnostic practices. Liebman and Burnette (2013), for example, found that clinicians were better able to identify BPD in women than in men, suggesting that the diagnosis remains more culturally legible when attached to femininity. This pattern may be reinforced by a research base that has historically centered white women, narrowing understandings of how BPD presents across genders and social locations (Masland et al. 2023). Valero et al. (2025) likewise found that women diagnosed with BPD reported invalidating experiences, including being pathologized for ordinary or socially recognizable forms of emotional distress. Labels such as “crazy” and “intense” appeared repeatedly in participants' accounts, underscoring the continuing association between femininity, excess emotion, and psychiatric discrediting (Valero et al. 2025). Shaw and Proctor (2005) similarly argue that the BPD label is often applied to women who express anger and aggression in ways that violate normative femininity.

Taken together, these historical and empirical threads suggest that BPD's association with women reflects the social and institutional construction of diagnostic meaning rather than prevalence rates alone. The diagnosis has developed through historical and institutional processes that render certain expressions of distress more recognizable as pathological when associated with women. As a result, emotional intensity, dependency, self‐harm, relational conflict, and perceived neediness continue to be interpreted through gendered assumptions that influence how BPD is understood and acted upon across contemporary systems of care, including nursing.

### 2.2 Historical Implications for Community Mental Health Nursing

Historical meanings of BPD are significant for CMHNs because the diagnosis rarely appears as a neutral label; it circulates through charts, referral histories, workplace norms, service pathways, and team language that already carry interpretive assumptions about what the diagnosis means. Within health care, BPD is often associated with manipulation, chronicity, treatment resistance, and high service use, assumptions that can reduce a person's credibility and narrow appropriate responses (Ma and Else‐Quest 2025; Navarre 2025; Ring and Lawn 2019). Requests for support may be interpreted as attention‐seeking rather than as indicators of serious distress (Cobbaert et al. 2026), and behavior may be interpreted through a negative diagnostic frame, with care withheld when providers assume a person is seeking attention rather than help (Sulzer 2015). Individuals diagnosed with BPD may consequently experience their concerns as dismissed, doubted, or attributed primarily to the diagnosis itself (Navarre 2025). Dickens et al. (2016) found that mental health nurses reported more negative attitudes, less empathy, and less treatment optimism toward individuals diagnosed with BPD than other professionals or other mental health diagnoses. Although such findings are often discussed in terms of individual bias, a genealogical reading suggests that these responses are also sustained by a longer history that marks certain forms of suffering as excessive, difficult, or undeserving. What appears as interpersonal stigma in the present is, in many cases, linked to inherited psychiatric meanings that continue to shape how distress is recognized and addressed.

For CMHN practice, this history has practical implications. Nursing ethics require safe, equitable, and non‐discriminatory care regardless of diagnosis (CNO 2026a), yet gendered and institutional meanings attached to BPD can influence what nurses notice, how behavior is interpreted, and which care options appear available. Attending to this history is therefore necessary to understand how psychiatry's gendered past continues to organize present care. The analysis that follows examines how these meanings become embedded in community mental health systems via policies, documentation practices, referral processes, and service pathways, and how that embedding shapes the organization, delivery, and location of care.

## 3 Organization of BPD in Mental Health Systems

### 3.1 Diagnostic Category of BPD and Organization of Health Services

Building on Tichenor et al. (2025), the analysis shifts from asking what BPD “really is” to examining what the diagnostic category does, for whom, within which institutional contexts, and toward what ends. Rather than treating BPD as a fixed medical entity, Tichenor et al. (2025) encourage attention to the social and political effects of the diagnosis and the ways these effects vary across contexts. This analysis extends that insight by examining how BPD becomes consequential through documentation, service eligibility, referral pathways, and treatment decisions, thereby organizing clinical work and shaping how nurses interpret people's experiences. Consistent with Tichenor et al.'s (2025) emphasis on what the diagnosis does rather than what it is, this analysis approaches BPD as a social relation and institutional category whose effects are produced through institutional practices and processes rather than as a fixed and enduring medical condition.

To understand how BPD comes to organize clinical work, it is first necessary to consider how the diagnosis is produced within psychiatric practice. BPD may be diagnosed during a clinical assessment when evidence is available to indicate that diagnostic criteria are met (APA 2022). Within the DSM‐5‐TR framework, diagnosis requires that at least five of nine criteria be met; however, the DSM‐5‐TR does not specify a minimum duration for symptoms, leaving substantial room for clinical judgment (APA 2022). As a result, diagnosis is not necessarily dependent on repeated presentations over time but may be established during a single clinical encounter. In addition, the diagnosis is not a purely objective process. It is shaped by clinician interpretation, cultural assumptions, available information, and institutional conditions such as time constraints, service pressures, and resource limitations. Once recorded, the diagnosis becomes part of the clinical record and travels across settings, informing later assessments, referrals, consultations, and care plans.

Beyond description, BPD is embedded within health service design; treatment recommendations influence where care occurs, which services are funded, how waitlists are managed, and what roles nurses assume. Although psychological therapies are recommended for BPD, such as dialectical behavioral therapy (DBT), pharmacological treatments remain widely used despite limited evidence for treating BPD pathology (Stoffers‐Winterling et al. 2022; Tennant et al. 2023). Given the limited role of medication, clinical guidelines emphasize psychological therapies, supportive interventions, and continuity of care in community settings (Keepers et al. 2024; National Institute for Health and Care Excellence 2009). Clinical guidelines are intended to enhance quality of care and reduce adverse outcomes; however, a significant gap remains between recommendations and practice. Cases et al. (2020), for example, found that individuals diagnosed with BPD who presented to emergency departments often experienced inconsistent referrals, long wait times, and limited follow‐up, contributing to repeated service use and discontinuity of care.

These organizational effects extend beyond treatment recommendations to include assessments of risk, service eligibility, prioritization, and resource allocation. While the diagnosis can be debilitating through stigma and exclusion, it can also be capacitating by enabling access to treatment, resources, validation, and collective advocacy (Tichenor et al. 2025). However, these effects are context‐dependent and shaped by relations of power, privilege, and marginalization. Presentations involving self‐harm or suicidality may be framed as chronic or expected rather than acute, influencing urgency of response, prioritization in emergency settings, and decisions about admission or discharge. In this sense, BPD functions not only as a classification of symptoms but also as an organizing mechanism that shapes access and treatment within the health care system.

### 3.2 Structural Stigma Shaping Health Care Systems

Structural stigma refers to “societal‐level conditions, cultural norms, and institutional policies that constrain the opportunities, resources, and well‐being of the stigmatized” (Hatzenbuehler 2016, 742). In practice, it is enacted through organizational and clinical decision‐making processes that position some forms of distress as chronic, unresponsive, or less urgent, shaping the availability and distribution of care for individuals with BPD. Access to effective therapies is often limited by cost, service availability, long waitlists, and shortages of clinicians trained to provide specialized treatment (Iliakis et al. 2019; Leichsenring et al. 2024). Fragmented health services, inadequate staffing, limited role clarity, and poor access to clear information further constrain access and may intensify stigmatizing health care encounters (Liegghio 2017; Livingston 2013). These conditions demonstrate that care is not allocated solely based on clinical need; rather, it is organized through decisions about resourcing, specialization, eligibility, and service design.

Structural stigma influences care delivery location. Long‐term hospitalization has often been viewed as inappropriate for individuals diagnosed with BPD (Paris 2004), contributing to a strong emphasis on community‐based care (O'Connell and Dowling 2013). While community care may be appropriate in many cases, this pattern is also shaped by assumptions about dependency, repeated readmission, risk, and resource allocation (Leichsenring et al. 2024). Emerging evidence suggests that brief admissions may provide benefit during periods of crisis, although their implementation remains inconsistent across services (Tan and Hope 2025). Refusal of hospital admission can generate significant anxiety for individuals diagnosed with BPD and may shift care to community services that are not always adequately resourced (Klein et al. 2026).

Diagnostic labels frequently determine program eligibility, referral decisions, and resource allocation; individuals with BPD may be excluded because they are viewed as treatment resistant, high service users, clinically complex, or unsuitable for certain programs (Klein et al. 2022). Interpersonal perceptions of individuals with BPD as “challenging” can interact with institutional constraints to influence decision‐making and service exclusion (Klein et al. 2022; McCarrick et al. 2022). In the context of BPD, these institutional logics can intensify structural stigma by privileging brief, measurable interventions and rendering sustained relational care difficult to justify for those positioned as complex or time intensive.

These institutional arrangements must also be situated within the political economy of contemporary public mental health care. Neoliberal reforms, marketization, austerity, and performance management privilege throughput, short‐term intervention, risk documentation, and quantifiable outcomes, often at the expense of sustained relational work (Moth 2023). Within these conditions, diagnostic categories can function as managerial tools that sort people according to perceived risk, treatability, and service suitability (Moth 2023; Rankin and Campbell 2006). Responsibilization further relocates institutionally produced problems onto individuals by positioning service users as responsible for their recovery and practitioners as individually accountable for care deficits arising from under‐resourced systems (Moth 2023). A Mad Studies analysis extends this political‐economic critique by examining whose knowledge informs institutional definitions of recovery, risk, and appropriate service use (Burstow 2015; LeFrançois et al. 2013; Redikopp 2018). From this perspective, privileging institutional knowledge over the experiential knowledge of people receiving care can obscure how service structures produce exclusion and instead position people diagnosed with BPD as the source of institutionally organized difficulties. Addressing these issues therefore requires attention not only to individual attitudes but also to the institutional conditions that determine which forms of care are possible, valued, and resourced.

## 4 Application in Community Mental Health Nursing Practice

In community settings, CMHNs commonly first encounter a BPD diagnosis through charts, referral histories, clinical documentation, and team communications. These records circulate across services, meaning that BPD is often encountered as an institutionalized category that shapes expectations before any direct patient interaction occurs.

The gendered and institutional meanings attached to BPD become embedded in routine nursing practices, including assessment, documentation, eligibility decisions, relational engagement, and care coordination. In doing so, they shape what is noticed, how behavior is interpreted, and which responses are considered appropriate. The following sections examine three domains through which BPD organizes CMHN practice: clinical visibility, relational care, and service access and continuity of care.

### 4.1 Clinical Visibility Under Diagnostic Conditions

Diagnostic categories influence not only how individuals are classified but also which aspects of their experience become clinically visible. Stroud and Parsons (2013) found that CMHNs' interpretations of patient behavior influenced their responses. When nurses were unable to ascribe meaning to a patient's actions, they were more likely to interpret them negatively. This tendency may be amplified when a diagnostic label primes clinicians to interpret crisis presentations, self‐harm, withdrawal, anger, or repeated service contact through assumptions associated with BPD rather than as contextual responses to distress.

Documentation practices can further narrow clinical visibility. Notes are carried forward and inform subsequent assessments, creating continuity in interpretation as well as care. The CNO (2026b) requires documentation to be clear, accurate, and non‐discriminatory; yet stigmatizing language in charts can prefigure clinicians' attitudes and decisions. Martin et al. (2020) identify risks associated with negative writing styles in clinical notes, finding that biased documentation can shape readers' interpretations of patients and encourage attribution of behavior primarily to mental illness. Such language may lead to dehumanizing assumptions and stigmatizing framing based on biased interpretations (Ma and Else‐Quest 2025). In later work, Martin et al. (2022) found that nurses who read biased notes were less likely to provide health teaching related to prescribed medications and more likely to select medication‐based interventions over educational responses.

These findings suggest that documentation does not merely record care; it can actively shape clinical judgment and intervention. Over time, particular understandings of behavior may become stabilized, making alternative explanations less likely to be considered. For individuals diagnosed with BPD, distress or crisis may come to be interpreted as expected or chronic features of the diagnosis rather than as indicators of acute need. Terms such as “BPD traits,” “chronic self‐harm,” or “attention‐seeking” can limit consideration of alternative explanations by framing behavior as predictable, manipulative, or less urgent (Ma and Else‐Quest 2025; Navarre 2025). Diagnostic visibility matters because the ways nurses make sense of patients' experiences inevitably shape how they engage with them. These interpretive processes extend beyond assessment and documentation into the formation and maintenance of therapeutic relationships.

### 4.2 Diagnosis Shaping Relational Care

At its best, CMHN is relational and longitudinal, developing through ongoing interactions over time. However, the institutional conditions of practice can constrain the realization of these relational commitments. Within this context, the diagnosis of BPD can influence the conditions under which therapeutic relationships are formed, particularly through its effects on trust, credibility, boundaries, and the perceived legitimacy of distress (McGrath and Dowling 2012). Diagnostic assumptions may contribute to therapeutic pessimism, heightened vigilance, concerns about dependency, or doubts about the credibility of patients' reports, all of which can affect the quality of engagement between nurses and patients (Navarre 2025; Ring and Lawn 2019). As a result, individuals diagnosed with BPD may experience their distress as doubted, minimized, or viewed as less credible, creating barriers to the development of trust and therapeutic alliance (Navarre 2025). While professional boundaries are an important component of nursing practice, tensions can arise when efforts to manage risk or maintain boundaries overshadow opportunities for relational engagement and collaborative care. Haslam et al. (2026) describe reciprocal insecurity in crisis encounters, whereby nurses and service users each seek safety under conditions of structural vulnerability, yet one party's protective actions may be experienced as threatening by the other. This connects emotional labor and relational tension to the institutional organization of care rather than attributing these dynamics solely to individual attitudes.

These relational dynamics can also affect nurses themselves. Balancing empathy with risk management, responding to repeated crises, and working within resource‐constrained systems can increase emotional labor and contribute to moral distress, burnout, and boundary tensions (Delgado et al. 2021; Galea et al. 2026). Such tensions may be particularly pronounced in community settings, where sustained therapeutic relationships are central to care. Lamb et al. (2012) describe a form of dissonance between health care providers and patients that reflects more than a gap in mental health literacy; it may also involve a reluctance or limited capacity to engage with patients' own understandings of themselves and their experiences. Boundary‐setting may help reduce practitioner burnout (Nordkamp et al. 2024), but when enacted rigidly or punitively it can be experienced as cold or dismissive, undermining trust and limiting opportunities for meaningful engagement.

Training gaps and insufficient clinical support may further reinforce these dynamics. Nurses frequently report frustration, uncertainty, and feelings of ineffectiveness when responding to BPD‐related crises, often because of inadequate education, guidance, and organizational support (Klein et al. 2022; McCarrick et al. 2022). Perceptions of patients as “difficult” may contribute to reduced engagement and exclusionary practices, while limited opportunities for education and support can make it more challenging for nurses to sustain therapeutic engagement. Black et al. (2011) found that additional education improved health care providers' understanding of the distress experienced by individuals diagnosed with BPD, suggesting that professional knowledge influences both attitudes and quality of care. These findings indicate that relational care is shaped not only by individual nurses' attitudes but also by the educational and organizational contexts in which care is delivered (Desai et al. 2021).

### 4.3 Service Access and Continuity of Care

Beyond shaping clinical visibility and relational engagement, a BPD diagnosis can also influence access to services and continuity of care within community mental health systems. CMHNs do not simply observe these pathways; they work within them, negotiating referrals, explaining exclusions, managing follow‐up, responding to crises, and attempting to sustain continuity across service transitions (Hegedüs et al. 2025; O'Connell and Dowling 2013; Sather et al. 2018). As a result, nursing practice is shaped by institutional arrangements that govern access and eligibility.

In Canada, primary health care providers often serve as the initial point of contact and referral pathway to specialized mental health services (Government of Canada 2026). Individuals without regular primary care or with fragmented patterns of care may encounter barriers related to low referral rates, inadequate screening, poor interprofessional collaboration, limited follow‐up, and a lack of transparency regarding available services (Karimi et al. 2022). Role ambiguity between providers can further complicate transitions across levels of care (Sather et al. 2018). For CMHNs, these gaps are experienced in practical terms through unclear referral expectations, incomplete information, delayed follow‐up, and difficulties coordinating care across multiple providers.

Referral decisions may also be shaped by implicit bias and diagnostic assumptions. When practitioners make referral decisions for patients with complex or stigmatized diagnoses, they may rely on stereotypes or normative assumptions in the absence of adequate support and clear guidance (Koekkoek et al. 2011). Practitioners may also prefer to work with patients perceived as easier to engage, creating barriers for stigmatized groups (Desai et al. 2021). These dynamics can contribute to patients' mistrust of health care providers and reluctance to seek support (Karimi et al. 2022). Primary care providers may function as gatekeepers to mental health services, and gatekeeping can become a barrier when access depends on provider knowledge, attitudes, availability, and referral practices (Mandangu et al. 2025). Community mental health nurses are often left to navigate the consequences of these gatekeeping processes, including delayed referrals, incomplete transitions, and repeated attempts to connect patients with appropriate services.

From a Mad Studies perspective, mistrust, refusal, or disengagement should not automatically be interpreted as evidence of insufficient insight, nonadherence, or unreadiness for treatment (Burstow 2015). These responses may instead reflect situated and experiential knowledge developed through encounters with coercion, invalidation, diagnostic overshadowing, exclusion, or psychiatric harm. Lester (2013) further argues that experiences commonly associated with BPD are often rooted in relational and social contexts and may represent attempts to survive, manage, or make sense of overwhelming distress rather than evidence of individual pathology. When these responses are interpreted through a pathological lens, individuals may be positioned as difficult, resistant, or unsuitable for care, reinforcing barriers to service access.

Eligibility criteria for specialized programs may further limit access. Individuals diagnosed with BPD may be excluded if they are unable to meet referral requirements, maintain ongoing therapy, demonstrate readiness for treatment, or align with specific program models. For example, some outpatient DBT programs require participants to have an existing therapist or external support network, potentially excluding those actively seeking help but lacking stable supports (Centre for Addiction and Mental Health 2026). Although such criteria may be intended to promote safety or program effectiveness, they may also reproduce inequities by privileging individuals who already possess resources, provider connections, and system‐navigation support. Access may be further constrained by service availability, geographic location, cost, waitlists, and limited program capacity. Even when referrals are successful, individuals may face delayed access, brief interventions, or discontinuous care that does not correspond to the chronic and fluctuating nature of their needs (Cases et al. 2020; Koekkoek et al. 2011). In these circumstances, CMHNs often work to maintain engagement, coordinate services, and support individuals during gaps in care.

Health service access is also influenced by assumptions about patient suitability, treatment engagement, and expected outcomes. Individuals diagnosed with BPD may be positioned as high service users, treatment resistant, or unlikely to benefit from care, contributing to inconsistent follow‐up and cyclical involvement across emergency, inpatient, and community settings. Although not specific to BPD, Goldman‐Mellor et al. (2020) found that patients presenting with intentional self‐harm had higher rates of emergency department use, inpatient admissions, and health care costs than psychiatric patients without self‐harm. Within the context of BPD, such findings may be interpreted as evidence of excessive service use; however, they may also reflect inadequate access to coordinated, sustained, and appropriate forms of care.

These patterns are reinforced by organizational conditions including limited program availability, long waitlists, staffing shortages, funding constraints, and insufficient coordination across providers. Gaps in training and inconsistent implementation of clinical guidelines can further restrict nurses' ability to provide responsive and sustained support (Iliakis et al. 2019). Health care professionals may also lack the organizational resources required to establish and maintain evidence‐based treatment programs, contributing to burnout and reduced job satisfaction among clinicians working with this population (King et al. 2018; Swenson et al. 2002). These findings suggest that access and continuity are not determined solely by patient need but are shaped by institutional arrangements that coordinate eligibility, resource allocation, and service delivery. For CMHNs, these conditions require ongoing efforts to support individuals across systems that may repeatedly fragment, defer, or restrict care.

## 5 Implications and Recommendations

The preceding analysis demonstrates that challenges associated with BPD in CMHN cannot be reduced to individual clinician attitudes alone. Because diagnostic meanings are reproduced through clinical, organizational, and institutional processes, responses must occur across multiple levels of practice. The following implications and recommendations address nursing practice, professional development, service delivery, and system reform.

### 5.1 Clinical Practice

At the practice level, CMHNs should adopt relational, trauma‐informed, and intersectional approaches that expand assessments beyond diagnostic assumptions. Patients report greater trust when nurses attend to their unique experiences rather than generalizing from a diagnosis (Romeu‐Labayen et al. 2020), and privileging diagnosis as identity may constrain recovery (Ng et al. 2019). Nurses should therefore approach BPD as one aspect of a person's broader life history rather than as the primary explanation for all behavior.

Trauma‐informed and intersectional approaches may further support more holistic assessment. Experiences of BPD and access to care are shaped by intersecting social locations and structural conditions (Duff et al. 2020; Ma and Else‐Quest 2025). Trauma‐informed care can support recovery by emphasizing safety, choice, collaboration, and attention to the possibility of re‐traumatization (Melillo et al. 2025). Similarly, nonjudgmental documentation can reduce the harms associated with biased clinical records. Healy et al. (2022) found that stigmatizing terminology in charts reinforced power imbalances between patients and providers. The use of labels perpetuates bias in their interactions, and these notions get passed on to future interactions with staff members (Healy et al. 2022). Nurses must be aware of these biases to avoid pathologizing the experiences of their patients. For CMHN, attention to language, context, and relational meaning can expand the scope of care beyond diagnostic assumptions and support more equitable responses to distress.

### 5.2 Education and Professional Development

Improving care for individuals diagnosed with BPD also requires strengthening nursing education and ongoing professional development. Education should move beyond disorder‐focused content to include lived‐experience perspectives and critical examination of how diagnostic categories shape clinical practice. Individuals diagnosed with BPD identify confidence, nonjudgmental engagement, and psychological safety as important qualities in nursing interactions (Romeu‐Labayen et al. 2022), highlighting the value of educational approaches that foster empathy and relational understanding.

Professional development initiatives should incorporate relational inquiry as a framework for practice. Relational inquiry encourages nurses to attend to social, structural, and interpersonal contexts, while reflecting critically on power, assumptions, and relational dynamics (Doane and Varcoe 2021). Education and training must also make structural stigma visible and create space for critical reflection on cultural humility (Ma and Else‐Quest 2025). This includes fostering reflexivity about how power, privilege, and inequity shape the production and reproduction of stigma, and supporting nurses to recognize their own roles within these processes (Liegghio 2017; Ma and Else‐Quest 2025). These principles align with relational inquiry, which emphasizes critical engagement with context and attention to the broader social forces shaping practice (Doane and Varcoe 2021). Nurses must consider the impact of both interpersonal and intrapersonal factors to inform their nursing practice (Younas 2020). Integrating these ideas into clinical practices can support nurses' ability to connect with patients on a deeper level and improve future interactions.

### 5.3 Organizational, Policy, and Health System Reform

Improving care for individuals diagnosed with BPD requires reforms at organizational, service delivery, and health system levels. Efforts should focus not only on creating clearer referral pathways but also on addressing the institutional arrangements that determine eligibility, prioritize particular forms of need, and shape access to services. Policies that support integrated care pathways, transparent eligibility criteria, collaborative decision‐making, and consistent follow‐up across sectors may promote more equitable access to care while reducing fragmentation and strengthening continuity across service transitions (Mandangu et al. 2025; Pourat et al. 2019).

Organizations and health systems should also invest in service structures that support innovative and alternative approaches to care. Recovery‐oriented and low‐barrier models offer promising alternatives to traditional crisis‐focused services. For example, Mortimer‐Jones et al. (2019) describe the Open Borders Programme, which combined brief admissions, respite care, and telephone coaching for individuals with BPD who were frequent users of public mental health services. Staff and service users reported benefits including flexible access to care, individualized support, stronger therapeutic relationships, reduced self‐harm, decreased hospitalization, and increased self‐efficacy. Similar rapid‐access community mental health services have been developed to improve service accessibility through prompt assessment, medication management, brief therapeutic interventions, and follow‐up care following emergency mental health presentations. These examples suggest that service models emphasizing accessibility, continuity, flexibility, and recovery may offer viable alternatives to fragmented systems of care.

These service innovations highlight the importance of designing systems that prioritize accessibility, continuity, and recovery. However, the effectiveness and sustainability of such models depend not only on service design but also on an adequately supported workforce. Collaborative care approaches that emphasize shared decision‐making, self‐management, and nurse‐led coordination have also demonstrated promise for individuals with severe personality disorders (Stringer et al. 2011). Expanding access to evidence‐informed treatments while reducing restrictive eligibility criteria may improve service accessibility and better align practice with existing clinical guidelines (Iliakis et al. 2019; NICE 2009). Importantly, reforms must also address workforce capacity and organizational sustainability. Increased access to training, clinical supervision, interprofessional collaboration, and workplace support may improve both patient outcomes and clinician well‐being (Delgado et al. 2021; Iliakis et al. 2019). In many helping professions, formal mechanisms such as clinical supervision are embedded within routine practice to support reflection, professional development, and the management of emotionally demanding clinical work (Keum and Wang 2021). Such approaches have also been identified as potentially beneficial for nurses by strengthening resilience and reducing moral distress (Mack 2021). Organizations should therefore invest in regular clinical supervision, mentorship opportunities, reflective practice initiatives, and workload supports that recognize the emotional labor associated with relational mental health care. These measures may help sustain therapeutic engagement, reduce burnout, and strengthen nurses' capacity to provide responsive and equitable care within resource‐constrained systems.

Policy frameworks should also promote accountability for equitable access to mental health services and ensure that diagnostic labels are not used to justify exclusion from care. Such frameworks should support adequate staffing, sustainable funding for community mental health services, and organizational structures that facilitate continuity of care across sectors and providers. These reforms recognize that challenges associated with BPD are not solely clinical concerns but are also shaped by the organization, funding, and governance of mental health systems. Strengthening organizational supports and investing in accessible, recovery‐oriented models of care may help CMHNs navigate fragmented systems while providing more equitable and responsive care for individuals diagnosed with BPD. However, reforms focused solely on improving existing mental health services may not fully address concerns raised by Mad Studies scholars regarding the role of psychiatry in organizing care and defining legitimate forms of support (Adam 2026; Johansson et al. 2024). Reform within existing systems should therefore be accompanied by investment in supports that are not organized primarily through psychiatric diagnosis or treatment compliance. This requires nurses to critically examine how everyday practices such as assessment, documentation, and risk management may reproduce psychiatric authority and stabilize psychiatric categories (Adam 2026). While reforms within existing systems remain important, Mad Studies scholars have also emphasized the need for supports that exist outside conventional psychiatric services, including peer‐led and survivor‐led initiatives, community crisis alternatives, and other forms of collective support (Adam 2026). Similarly, Johansson et al. (2024) argue that outreach nurses should move beyond approaches grounded exclusively in psychiatric and biomedical knowledge and instead form alliances with disaffiliated populations in ways that resist co‐optation and respect their self‐determined priorities. Rather than seeking to reintegrate individuals into psychiatric systems, nurses might experiment with alternative, relationship‐based forms of engagement that honor refusal, support autonomy, and create space for approaches to wellness defined by those receiving care.

## 6 Limitations and Future Directions

This paper is a critical conceptual analysis and therefore has several limitations. It does not present original empirical data. While this approach allows for a broad and theoretically informed analysis, it is limited by the scope, quality, and underlying assumptions of the available literature. The central focus of the paper is women's experiences, reflecting both the gendered nature of the BPD diagnosis and the predominance of cisgender women within the existing research base. However, this focus also reflects a limitation within the literature itself: the relative absence of attention to gender‐diverse populations, including transgender, non‐binary, and Two‐Spirit people (Denning et al. 2022). The experiences of these groups in relation to BPD diagnosis, stigma, and care remain underexplored, and it is not yet well understood whether the diagnosis operates similarly or differently across diverse gender identities. Emerging scholarship suggests that gender‐diverse individuals may experience higher rates of BPD diagnosis (Denning et al. 2022; Rodriguez‐Seijas et al. 2024), and that exploration of gender identity may itself be pathologized within psychiatric contexts (Seal et al. 2026). These findings point to an important area for future research.

The existing literature on BPD is limited in its engagement with race and ethnicity. Much of the research has been conducted with predominantly white populations or fails to report race and ethnicity altogether. Yet variation in symptom prevalence, interpretation, and experience across cultural contexts has been recognized for more than a decade (e.g., Jani et al. 2016). As a result, this analysis is constrained in its ability to examine how racialization and colonization intersect with gender to shape the diagnosis and care of BPD. This gap has important implications for culturally safe, affirming, and responsive care.

Finally, this paper cannot fully account for variation across organizational settings, geographic contexts, funding models, and professional roles. For example, experiences of care may differ substantially between publicly funded health systems and services that rely on private insurance or out‐of‐pocket payment, where eligibility criteria, service availability, and treatment options may be shaped by different financial and organizational structures.

Future research on CMHN is needed to examine how diagnostic meanings are taken up, resisted, or reproduced in specific practice settings. Such work should attend to documentation practices, referral processes, eligibility criteria, clinical supervision, interprofessional collaboration, and access to specialized services. Future research should examine what nurses need in practice to critically engage diagnostic categories, recognize structural and gendered assumptions, and respond to distress in ways that are relational, contextual, and equitable. Incorporating insights from Mad studies may further strengthen this work by foregrounding lived experience, challenging pathologizing frameworks, and re‐centering questions of power, voice, and resistance within mental health care. An intersectional approach is particularly important for examining how the BPD diagnosis shapes access to care and health resources across different social locations. Future research should examine the varied consequences of the diagnosis and the ways these consequences are differentially produced and distributed through relations of power, privilege, and marginalization (Tichenor et al. 2025).

## 7 Conclusion

In summary, this paper examined the historical and institutional genealogical development of the BPD diagnosis. Using critical theory perspectives, including feminist and mad studies approaches, BPD was explored as a systematic categorization of non‐normative or non‐desirous gendered behaviors into symptoms of mental disorder. This categorization shapes the institutional processes informing CMHN practice through managerial systems designed to organize and dictate clinical treatment, rather than through trauma‐informed relational inquiry and client‐centered nursing care. The persistence of structural stigma, informed by historical patterns, is demonstrated through the practices and work processes inherent in the health care system, including in the type and quality of resources available. Diagnostic assumptions and system‐level conditions and constraints create gaps in access to recommended treatment and equitable care. The BPD label and diagnostic criteria that define eligibility for treatment programs can also be used as cause for limitation or exclusion from participation.

Clinical visibility, relational care, and continuity of services were examined as domains of CMHN practice organization. Various forms of nursing documentation act as sources for the formation of attitudes, perceptions, and interpretations of patients diagnosed with BPD, resulting in preconceived expectations. Relational care, with contextual, trauma‐informed, and intersectional approaches, relocates the BPD diagnosis away from the primary focus of care. However, the biases of health care providers and the stigmatization that accompanies the BPD diagnosis impact referral decisions and program availability and often negate the needs of individual patients.

To address the systemic categorization of BPD, action must be taken across multiple levels, including clinical practice, education and professional development, and organizational, policy, and health system reform. CMHNs must focus care on the context of the individual patient's circumstances and experiences, and not on clinical diagnosis alone. Nursing education and professional development must continue to shift toward relational inquiry and lived experience. CMHN must advocate for institutional arrangements and managerial communication tools that support equitable, consistent, and flexible care, recovery‐oriented models, and innovative systems designs. Finally, collaborative care approaches, with improved workplace capacity and organizational sustainability, will strengthen the resilience and reduce the moral distress experienced by CMHNs, thus supporting their provision of equitable and responsive care.

## Author Contributions

Rachel Galea conceived the manuscript, conducted the literature review, and led manuscript writing and revision. Alison Rodgers contributed to manuscript writing and critical revisions. Catherine MacDonald contributed to manuscript review and revisions. Ayanna Robertson and Alessandra Rooney contributed to literature review activities, manuscript development, and revisions as part of a community nursing clinical placement. All authors reviewed and approved the final manuscript.

## Funding

The authors have nothing to report.

## Ethics Statement

The authors have nothing to report.

## Conflicts of Interest

The authors declare no conflicts of interest.

## Disclosing the Use of Artificial Intelligence

The authors confirm that the generative artificial intelligence (AI) tool, Microsoft Co‐Pilot, was used to assist with manuscript editing and structural revision during preparation of this manuscript. All intellectual content and theoretical arguments presented in the manuscript remain solely those of the authors.

## Data Availability

The authors have nothing to report.
